# 1,4-Bis(1,1-dimethyl­prop­yl)-2,5-dimeth­oxy­benzene

**DOI:** 10.1107/S160053681103772X

**Published:** 2011-09-30

**Authors:** Kiichi Amimoto

**Affiliations:** a Department of Science Education, Graduate School of Education, Hiroshima University, 1-1-1 Kagamiyama, Higashi–Hiroshima, Hiroshima, Japan

## Abstract

The title compound, C_18_H_30_O_2_, was prepared by Friedel–Crafts alkyl­ation of 1,4-dimeth­oxy­benzene with 2-methyl-2-butanol. The complete mol­ecule is generated by the application of a crystallographic centre of inversion. The two meth­oxy groups are oriented in the same plane of the aromatic ring [C—C—O—C torsion angle = 9.14 (16)°]. While one methyl group of the *tert*-pentyl substituent is coplanar with the benzene ring [C—C—C—C = 0.45 (15)°] and lies towards the less-hindered H atom, the other methyl and ethyl groups are directed to either side of the benzene ring [C—C—C—C torsion angles = 118.78 (12) and 59.11 (14)°, respectively]. In the crystal, the hydro­phobic mol­ecules pack to form a brick-wall-like architecture.

## Related literature

For the synthesis of the title compound, see: Polito *et al.* (2010 ▶) and for the synthesis of the analogous compound, 1,4-di-*tert*-butyl-2,5-dimeth­oxy­benzene, see: Williamson *et al.* (2006 ▶). For the unique crystal growth of 1,4-di-*tert*-butyl-2,5-dimeth­oxy­benzene, see: Blatchly & Hartshorne (1966 ▶). For the crystal structure of 1,4-di-*tert*-butyl-2,5-dimeth­oxy­benzene, see: Rosokha & Kochi (2007 ▶).
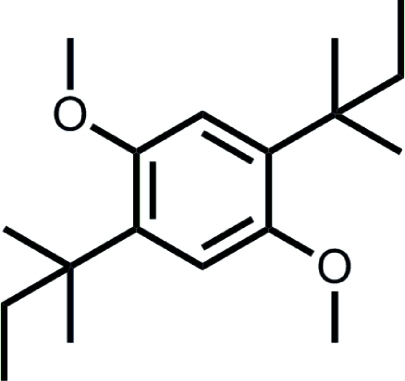


## Experimental

### 

#### Crystal data

C_18_H_30_O_2_
*M* *_r_* = 278.42
Triclinic, 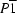

*a* = 6.456 (5) Å
*b* = 6.551 (5) Å
*c* = 10.800 (5) Å
α = 93.120 (5)°
β = 105.950 (5)°
γ = 108.460 (5)°
*V* = 411.5 (5) Å^3^
*Z* = 1
Mo *K*α radiation
μ = 0.07 mm^−1^
*T* = 90 K
0.4 × 0.2 × 0.1 mm

#### Data collection

Bruker APEXII CCD area-detector diffractometer
Absorption correction: empirical (using intensity measurements) (*SADABS*; Bruker, 2009 ▶) *T*_min_ = 0.972, *T*_max_ = 0.993
2473 measured reflections
1888 independent reflections
1698 reflections with *I* > 2σ(*I*)
*R*_int_ = 0.013

#### Refinement

*R*[*F* ^2^ > 2σ(*F* ^2^)] = 0.042
*wR*(*F* ^2^) = 0.114
*S* = 1.10
1888 reflections
151 parameters
All H-atom parameters refined
Δρ_max_ = 0.40 e Å^−3^
Δρ_min_ = −0.22 e Å^−3^

### 

Data collection: *APEX2* (Bruker, 2009 ▶); cell refinement: *SAINT* (Bruker, 2009 ▶); data reduction: *SAINT*; program(s) used to solve structure: *SIR97* (Altomare *et al.*, 1999 ▶); program(s) used to refine structure: *SHELXL97* (Sheldrick, 2008 ▶); molecular graphics: *Mercury* (Macrae *et al.*, 2008 ▶); software used to prepare material for publication: *WinGX* (Farrugia, 1999 ▶) and *publCIF* (Westrip, 2010 ▶).

## Supplementary Material

Crystal structure: contains datablock(s) I, global. DOI: 10.1107/S160053681103772X/tk2787sup1.cif

Structure factors: contains datablock(s) I. DOI: 10.1107/S160053681103772X/tk2787Isup2.hkl

Supplementary material file. DOI: 10.1107/S160053681103772X/tk2787Isup3.cml

Additional supplementary materials:  crystallographic information; 3D view; checkCIF report

## Figures and Tables

**Fig. 1.**
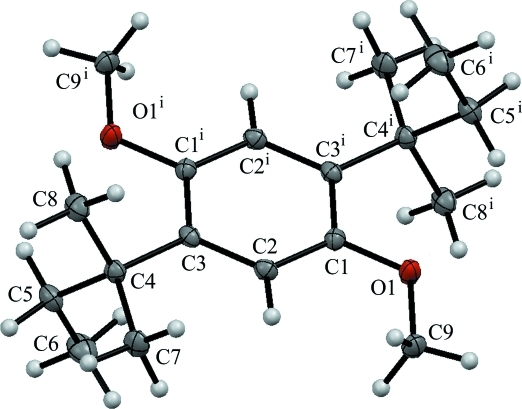
The molecular structure of the title compound, showing the atom-numbering scheme and displacement ellipsoids at the 50% probability level for non-H atoms. Atoms marked with i are at the symmetry positions 1 - x, 1 - y, 1 - z.

**Fig. 2.**
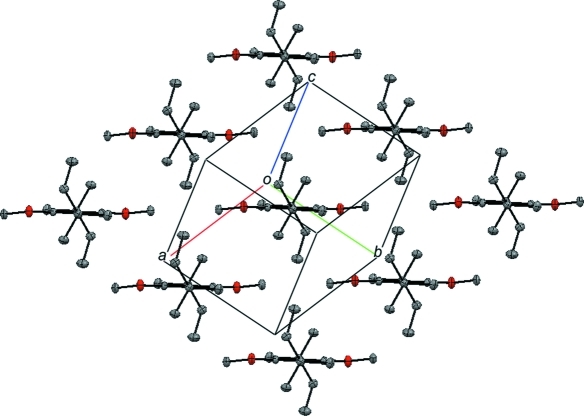
The crystal packing of the title compound showing the brick-wall packing arrangement. All H atoms are omitted for clarity.

## supplementary crystallographic information

### Comment

Electrophilic aromatic substitution is one of the key reactions in organic chemistry. The Friedel-Crafts alkylation of 1,4-dimethoxybenzene with *tert*-butyl alcohol is a popular introductory organic laboratory experiment to illustrate the features of electrophilic aromatic substitution (Williamson *et al.*, 2006). The product, 1,4-di-*tert*-butyl-2,5-dimethoxybenzene, (I), has also been reported to exhibit a dramatic change of shape during crystal growth (Blatchly and Hartshorne, 1966). Recently, a synthesis involving electrophilic aromatic substitution coupled with a Wagner-Meerwein rearrangement using 1,4-dimethoxybenzene with 2-methyl-2-butanol was reported (Polito *et al.*, 2010). However, the product, 1,4-bis(1,1-dimethylpropyl)-2,5-dimethoxybenzene (II), has no dramatic crystal growth behaviour. To obtain the perspectives on both development of sophisticated laboratory activity in organic chemistry and the relationship between crystal growth and molecular structure, the X-ray diffraction analysis of the title compound (II) was performed, and the structural features of (I) and (II) discussed in this article. The crystal structure of (I) has already been reported (Rosokha and Kochi, 2007).

Compound (I) crystallizes in the monoclinic space group *P*2_1_/*c*. The methoxy groups are inclined at 33.9 (1) ° to the benzene plane. The methyl C—H of the methoxy group points to the adjacent benzene ring in a face-on-edge manner, in which the distance between the methyl carbon and the π-plane is 3.47 (1) Å. The crystal packing exhibits a herringbone structure. Compound (II) crystallizes in the triclinic space group *P*1 (Fig. 1). The asymmetric unit of (II) contains half a molecule with the complete molecule being generated by a centre of inversion located in the benzene ring. In contrast to (I), the methoxy groups lie on almost the same plane as the benzene ring, where the dihedral angle is 4.9 (1) °. The ethyl chains of two *tert*-pentyl groups stand to one side of the benzene plane in an *anti*-orientation, where the dihedral angle is 83.8 (2) °. Since the ethyl chain and methyl group of *tert*-pentyl group and methoxy group are aggregated by hydrophobic interactions, each molecule is closely arranged in the brick-wall fashion (Fig. 2). Compared to (II) the packing mode of (I) seems to be relatively loose, resulting the expression of dynamic crystal growth of (I).

### Experimental

In a 50-ml Erlenmeyer flask were placed 690 mg (5 mmol) of 1,4-dimethoxybenzene, 1.3 g (1.5 mmol) of 2-methyl-2-butanol, and 1.5 ml of glacial acetic acid. The Erlenmeyer flask was immersed in an ice-water bath to cool the solution below 278 K. Concentrated sulfuric acid (5 ml) was added dropwise into the vigorously stirred reaction mixture so as not to exceed the temperature of 283 K. When all the sulfuric acid was added, the mixture was stirred at room temperature for 10 minutes. The Erlenmeyer flask was immersed again in an ice-water bath, and then a sufficient amount of ice-cold water was added into the reaction mixture to quench the reaction and isolate the product. The white solid was filtered off on a small Büchner funnel and washed with a small amount of ice-cold ethanol. Single crystals were obtained by recrystallization from its ethanol solution; *M*.pt. 382–383 K. ^1^H NMR (500 MHz, CDCl_3_):δ 6.72 (s, 2 H), 3.76 (s, 6 H), 1.77 (q, 4 H), 1.29 (s, 12 H), 0.62 (q, 6 H) p.p.m. IR (KBr): 2962, 2933, 2868, 1506, 1481, 1392, 1369, 1209, 1128, 1041, 865 cm^-1^. APCI-MS: *m/z* [M—H]^+^ = 277.21621.

### Refinement

All H atoms were found in a difference Fourier map and refined isotropically.

### Crystal data

**Table:**

C_18_H_30_O_2_	*Z* = 1
*M**_r_* = 278.42	*F*(000) = 154
Triclinic, *P*1	*D*_x_ = 1.123 Mg m^−^^3^
Hall symbol: -P 1	Mo *K*α radiation, λ = 0.71069 Å
*a* = 6.456 (5) Å	Cell parameters from 1654 reflections
*b* = 6.551 (5) Å	θ = 3.3–28.8°
*c* = 10.800 (5) Å	µ = 0.07 mm^−^^1^
α = 93.120 (5)°	*T* = 90 K
β = 105.950 (5)°	Plate, colourless
γ = 108.460 (5)°	0.4 × 0.2 × 0.1 mm
*V* = 411.5 (5) Å^3^	

### Data collection

**Table:**

Bruker APEXII CCD area-detector diffractometer	1888 independent reflections
Radiation source: fine-focus sealed tube	1698 reflections with *I* > 2σ(*I*)
graphite	*R*_int_ = 0.013
Detector resolution: 8.333 pixels mm^-1^	θ_max_ = 29.0°, θ_min_ = 2.0°
φ and ω scan	*h* = −8→5
Absorption correction: empirical (using intensity measurements) (*SADABS*; Bruker, 2009)'	*k* = −8→8
*T*_min_ = 0.972, *T*_max_ = 0.993	*l* = −12→14
2473 measured reflections	

### Refinement

**Table:**

Refinement on *F*^2^	Primary atom site location: structure-invariant direct methods
Least-squares matrix: full	Secondary atom site location: difference Fourier map
*R*[*F*^2^ > 2σ(*F*^2^)] = 0.042	Hydrogen site location: inferred from neighbouring sites
*wR*(*F*^2^) = 0.114	All H-atom parameters refined
*S* = 1.10	*w* = 1/[σ^2^(*F*_o_^2^) + (0.0566*P*)^2^ + 0.1363*P*] where *P* = (*F*_o_^2^ + 2*F*_c_^2^)/3
1888 reflections	(Δ/σ)_max_ < 0.001
151 parameters	Δρ_max_ = 0.40 e Å^−^^3^
0 restraints	Δρ_min_ = −0.22 e Å^−^^3^

### Special details

**Table:**

Geometry. All e.s.d.'s (except the e.s.d. in the dihedral angle between two l.s. planes) are estimated using the full covariance matrix. The cell e.s.d.'s are taken into account individually in the estimation of e.s.d.'s in distances, angles and torsion angles; correlations between e.s.d.'s in cell parameters are only used when they are defined by crystal symmetry. An approximate (isotropic) treatment of cell e.s.d.'s is used for estimating e.s.d.'s involving l.s. planes.
Refinement. Refinement of *F*^2^ against ALL reflections. The weighted *R*-factor *wR* and goodness of fit *S* are based on *F*^2^, conventional *R*-factors *R* are based on *F*, with *F* set to zero for negative *F*^2^. The threshold expression of *F*^2^ > σ(*F*^2^) is used only for calculating *R*-factors(gt) *etc*. and is not relevant to the choice of reflections for refinement. *R*-factors based on *F*^2^ are statistically about twice as large as those based on *F*, and *R*- factors based on ALL data will be even larger.

### Fractional atomic coordinates and isotropic or equivalent isotropic displacement parameters (Å^2^)

**Table:**

	*x*	*y*	*z*	*U*_iso_*/*U*_eq_	
C1	0.66045 (17)	0.40800 (16)	0.56010 (10)	0.0142 (2)	
C2	0.54020 (18)	0.34722 (16)	0.42749 (10)	0.0146 (2)	
C3	0.37620 (17)	0.43475 (16)	0.36311 (10)	0.0134 (2)	
C4	0.24450 (18)	0.36052 (16)	0.21713 (10)	0.0149 (2)	
C5	0.28316 (19)	0.55446 (18)	0.13993 (10)	0.0186 (2)	
C6	0.5309 (2)	0.7013 (2)	0.16805 (13)	0.0273 (3)	
C7	0.3162 (2)	0.18507 (18)	0.15790 (11)	0.0199 (3)	
C8	−0.01503 (19)	0.25829 (19)	0.19763 (11)	0.0203 (3)	
C9	0.8811 (2)	0.17879 (18)	0.54390 (11)	0.0191 (2)	
O1	0.81796 (14)	0.31572 (13)	0.62220 (7)	0.0203 (2)	
H2	0.574 (3)	0.240 (2)	0.3790 (14)	0.025 (4)*	
H5A	0.215 (3)	0.492 (2)	0.0462 (15)	0.023 (4)*	
H5B	0.196 (2)	0.646 (2)	0.1572 (14)	0.021 (3)*	
H6A	0.599 (3)	0.782 (3)	0.2639 (17)	0.036 (4)*	
H6B	0.635 (3)	0.615 (3)	0.1542 (17)	0.037 (4)*	
H6C	0.544 (3)	0.819 (3)	0.1069 (16)	0.034 (4)*	
H7A	0.226 (3)	0.138 (2)	0.0662 (15)	0.025 (4)*	
H7B	0.479 (3)	0.238 (3)	0.1626 (16)	0.030 (4)*	
H7C	0.286 (2)	0.050 (2)	0.2014 (14)	0.023 (3)*	
H8A	−0.073 (3)	0.360 (2)	0.2338 (14)	0.021 (3)*	
H8B	−0.046 (3)	0.123 (3)	0.2390 (15)	0.028 (4)*	
H8C	−0.097 (3)	0.216 (2)	0.1031 (15)	0.024 (4)*	
H9A	0.744 (3)	0.043 (2)	0.5000 (14)	0.024 (4)*	
H9B	1.000 (3)	0.144 (2)	0.6036 (15)	0.027 (4)*	
H9C	0.940 (2)	0.256 (2)	0.4774 (14)	0.021 (3)*	

### Atomic displacement parameters (Å^2^)

**Table:**

	*U*^11^	*U*^22^	*U*^33^	*U*^12^	*U*^13^	*U*^23^
C1	0.0147 (5)	0.0146 (5)	0.0137 (5)	0.0060 (4)	0.0038 (4)	0.0032 (4)
C2	0.0169 (5)	0.0136 (5)	0.0134 (5)	0.0052 (4)	0.0052 (4)	0.0005 (4)
C3	0.0140 (5)	0.0128 (5)	0.0117 (5)	0.0025 (4)	0.0039 (4)	0.0012 (3)
C4	0.0161 (5)	0.0155 (5)	0.0113 (5)	0.0049 (4)	0.0027 (4)	0.0001 (4)
C5	0.0227 (6)	0.0191 (5)	0.0127 (5)	0.0061 (4)	0.0046 (4)	0.0025 (4)
C6	0.0246 (6)	0.0294 (6)	0.0248 (6)	0.0034 (5)	0.0089 (5)	0.0072 (5)
C7	0.0241 (6)	0.0199 (5)	0.0138 (5)	0.0087 (4)	0.0024 (4)	−0.0029 (4)
C8	0.0168 (5)	0.0222 (6)	0.0166 (5)	0.0028 (4)	0.0020 (4)	0.0005 (4)
C9	0.0215 (5)	0.0214 (5)	0.0179 (5)	0.0129 (4)	0.0056 (4)	0.0021 (4)
O1	0.0255 (4)	0.0258 (4)	0.0137 (4)	0.0173 (3)	0.0028 (3)	0.0007 (3)

### Geometric parameters (Å, °)

**Table:**

C1—O1	1.3812 (14)	C6—H6A	1.043 (17)
C1—C2	1.3941 (15)	C6—H6B	1.037 (17)
C1—C3^i^	1.4051 (15)	C6—H6C	1.038 (18)
C2—C3	1.3988 (16)	C7—H7A	0.976 (15)
C2—H2	0.963 (15)	C7—H7B	0.984 (17)
C3—C1^i^	1.4051 (15)	C7—H7C	1.012 (16)
C3—C4	1.5371 (15)	C8—H8A	0.973 (15)
C4—C7	1.5376 (16)	C8—H8B	1.003 (17)
C4—C8	1.5428 (19)	C8—H8C	0.990 (15)
C4—C5	1.5494 (17)	C9—O1	1.4229 (14)
C5—C6	1.5172 (19)	C9—H9A	1.014 (16)
C5—H5A	0.991 (15)	C9—H9B	0.955 (16)
C5—H5B	0.985 (15)	C9—H9C	0.992 (15)
			
O1—C1—C2	121.95 (10)	H6A—C6—H6B	107.7 (13)
O1—C1—C3^i^	117.05 (9)	C5—C6—H6C	110.9 (9)
C2—C1—C3^i^	121.00 (10)	H6A—C6—H6C	107.8 (13)
C1—C2—C3	122.99 (10)	H6B—C6—H6C	107.0 (13)
C1—C2—H2	117.7 (9)	C4—C7—H7A	109.6 (9)
C3—C2—H2	119.3 (9)	C4—C7—H7B	112.6 (9)
C2—C3—C1^i^	116.02 (10)	H7A—C7—H7B	107.7 (13)
C2—C3—C4	121.35 (9)	C4—C7—H7C	112.1 (8)
C1^i^—C3—C4	122.63 (9)	H7A—C7—H7C	106.2 (12)
C3—C4—C7	111.63 (9)	H7B—C7—H7C	108.4 (12)
C3—C4—C8	109.87 (9)	C4—C8—H8A	111.4 (9)
C7—C4—C8	106.83 (9)	C4—C8—H8B	109.7 (9)
C3—C4—C5	111.30 (9)	H8A—C8—H8B	110.2 (13)
C7—C4—C5	108.72 (10)	C4—C8—H8C	108.6 (9)
C8—C4—C5	108.33 (9)	H8A—C8—H8C	108.9 (12)
C6—C5—C4	115.63 (10)	H8B—C8—H8C	107.9 (13)
C6—C5—H5A	110.5 (9)	O1—C9—H9A	109.7 (9)
C4—C5—H5A	106.6 (9)	O1—C9—H9B	104.8 (9)
C6—C5—H5B	107.9 (9)	H9A—C9—H9B	111.0 (12)
C4—C5—H5B	109.7 (9)	O1—C9—H9C	111.1 (8)
H5A—C5—H5B	106.2 (12)	H9A—C9—H9C	110.1 (12)
C5—C6—H6A	111.1 (10)	H9B—C9—H9C	110.1 (13)
C5—C6—H6B	112.1 (10)	C1—O1—C9	118.00 (9)

Symmetry codes: (i) −*x*+1, −*y*+1, −*z*+1.
